# Chronic Heat Stress Is Associated with Brain Injury, Blood–Brain Barrier Impairment, and Neuroinflammatory Gene Expression in Broilers

**DOI:** 10.3390/vetsci13040405

**Published:** 2026-04-20

**Authors:** Siliang Feng, Chenyang Zhou, Yajin Tie, Zhanqin Zhao, Mengyun Li, Lifang Si

**Affiliations:** College of Animal Science and Technology, Henan University of Science and Technology, Luoyang 471023, China; 230320181016@stu.haust.edu.cn (S.F.); 230320181062@stu.haust.edu.cn (C.Z.); 240320181071@stu.haust.edu.cn (Y.T.); zhaozhanqin@126.com (Z.Z.); mengyun.li@163.com (M.L.)

**Keywords:** heat stress (HS), oxidative stress (OS), brain injury, blood–brain barrier (BBB)

## Abstract

This study investigated the effects of chronic heat stress (HS) on brain injury in broilers and the associated molecular changes. A chronic HS model was established by exposing broilers to 35 °C from 08:00 to 20:00 daily from 21 to 42 days of age, and samples were collected at 28, 35, and 42 days of age. Chronic HS significantly impaired growth performance and was associated with histopathological and ultrastructural alterations in brain tissue. Serum antioxidant enzyme activities and the total antioxidant capacity were significantly reduced, whereas malondialdehyde levels were significantly increased, indicating sustained oxidative stress (OS). Blood–brain barrier (BBB) permeability, assessed by Evans blue extravasation, was significantly higher in HS birds and was accompanied by reduced mRNA expression of the tight junction-related genes ZO-1 and Claudin-5. In addition, chronic HS was associated with increased mRNA expression in genes related to cellular stress, oxidative stress, and inflammation, including key components of the TLR4/MyD88/NF-κB/NLRP3 pathway, as well as decreased expression of IL-4. These findings suggest that chronic HS is associated with enhanced OS, altered neuroinflammatory gene expression, and BBB impairment in the broiler brain. Overall, this study provides evidence that chronic HS is associated with brain injury in broilers and highlights a potential link among OS, inflammation-related transcriptional changes, and BBB dysfunction, thereby offering a basis for further mechanistic and interventional studies.

## Simple Summary

Heat stress (HS) is a common environmental challenge in broiler production that can negatively affect brain health. In this study, chronic HS reduced growth performance and was associated with histopathological brain damage, oxidative stress, and impaired blood–brain barrier (BBB) function. In the brain, HS increased the expression of genes linked to oxidative stress and inflammation, including key components of the TLR4/MyD88/NF-κB/NLRP3 pathway, and altered the expression of microglia-related genes. These findings suggest that chronic HS is associated with brain injury in broilers, possibly through coordinated changes in oxidative status, neuroinflammatory responses, and BBB integrity. This study provides useful insights into heat stress-related brain injury and may help to inform strategies to improve broiler health and productivity.

## 1. Introduction

As global climate change intensifies, the frequency and severity of extreme heat events are increasing, posing major challenges to intensive livestock farming. Broiler chickens, due to their high metabolic rate, lack of functional sweat glands, and dense plumage, have limited thermoregulatory capacity and are highly susceptible to heat stress (HS) under elevated ambient temperatures [1,2]. HS is known to impair feed intake, growth performance, and survival, and has been associated with pathological damage to the central nervous system (CNS) [3]. However, compared with studies on the liver, intestine, and heart, the effects of chronic HS on brain injury in broilers remain insufficiently understood.

The brain is one of the most metabolically active organs and depends on a tightly regulated internal environment to maintain normal function [4]. HS has been reported to induce mitochondrial dysfunction and disrupt antioxidant defenses, thereby exacerbating oxidative stress (OS) [5]. Because of its high oxygen consumption, abundance of polyunsaturated fatty acids, and relatively low antioxidant enzyme activity [6], the brain is particularly vulnerable to OS and thermal injury. Excessive reactive oxygen species (ROS) generation under HS may damage lipids, proteins, and nucleic acids [7,8]. In this context, antioxidant enzyme activities, the total antioxidant capacity (T-AOC), and lipid peroxidation-related indices are useful indicators for evaluating the oxidative status associated with HS-associated brain injury [9].

Heat shock proteins (HSPs) are highly conserved molecular chaperones induced in response to HS, OS, and other cellular stressors [10]. Among them, HSP27, HSP70, and HSP90 are closely associated with stress adaptation and inflammatory regulation. HSP27 is highly expressed in microglia under HS conditions [11] and may limit OS and apoptosis following neuronal or glial injury [12,13]. HSP70 is markedly induced during HS and has been implicated in protection against severe stress injury [14]. HSP90 has also been associated with inflammatory regulation [15], and HSP90α in particular has been linked to microglial pro-inflammatory responses [16,17]. Because HSPs may function not only as stress-response molecules but also as endogenous damage-associated molecular patterns (DAMPs), they are of particular interest in studies on HS-related neuroinflammation [18,19].

Microglia are the resident immune cells of the brain and play essential roles in immune surveillance and inflammatory responses [20]. Although microglial states are often broadly classified as pro-inflammatory and anti-inflammatory phenotypes [21,22], the present study mainly focused on activation-related changes, rather than the definitive characterization of polarization status [23]. Under chronic stress and tissue injury, activated microglia can release pro-inflammatory mediators such as tumor necrosis factor-α (TNF-α) and interleukin-1β (IL-1β), thereby contributing to neuroinflammation and tissue injury [24].

Among inflammatory pathways that are relevant to poultry, the Toll-like receptors (TLRs)/myeloid differentiation factor 88 (MyD88) and Nuclear Factor κ-light-chain-enhancer of activated B cells’ (NF-κB) pathways are considered to be particularly important [25,26]. Under chronic HS conditions, damaged brain tissue may release DAMPs such as HSP70 and HSP60 [27,28], which can be recognized by TLRs on the surface of microglia and thereby trigger downstream pro-inflammatory signaling [19,26,27]. TLR activation is primarily mediated through the MyD88-dependent pathway, which in turn activates NF-κB and promotes the transcription of inflammatory mediators, including TNF-α, IL-1β, and interleukin-6 (IL-6) [28]. NF-κB also promotes the transcription of inflammation-associated genes such as the NOD-like receptor family pyrin domain-containing protein 3 (NLRP3) [29]. NLRP3 can assemble with apoptosis-associated speck-like protein (ASC) and pro-Caspase-1 to form the NLRP3 inflammasome [30,31], which is involved in IL-1β and interleukin-18 (IL-18) maturation and inflammatory amplification [32,33,34]. However, whether chronic HS in broilers is associated with coordinated changes in TLR4/MyD88/NF-κB/NLRP3-related gene expression together with blood–brain barrier (BBB) impairment remains unclear.

The homeostasis of the CNS is largely maintained by the integrity of the BBB [35,36]. The BBB is widely viewed as a functional unit, termed the neurovascular unit, which consists of endothelial cells, pericytes, and astrocytic end-feet and is dynamically regulated by the adjacent neurons and microglia [37]. Its selective permeability depends largely on tight junctions between endothelial cells [38]. Among these, zonula occludens-1 (ZO-1) and Claudin-5 are critical for maintaining BBB integrity [39,40]. Increasing evidence suggests that inflammatory mediators and oxidative injury can disrupt tight junction organization and impair BBB function [39]. However, the relationship among chronic HS, inflammation-related gene expression, and BBB damage in broiler brain tissue has not been fully clarified.

Therefore, we hypothesized that chronic HS would be associated with brain injury in broilers, accompanied by enhanced OS, increased expression of TLR4/MyD88/NF-κB/NLRP3 pathway-related genes, and impairment of BBB integrity. Accordingly, the present study aimed to investigate the effects of chronic HS on brain injury in broilers and to characterize the associated changes in OS, inflammation-related gene expression, and BBB-related indicators.

## 2. Materials and Methods

### 2.1. Experimental Design

A total of 120 one-day-old male white-feathered broilers were obtained from a commercial hatchery and reared under controlled environmental conditions (25 ± 1 °C, relative humidity 60%) until 21 days of age, during which standard vaccination procedures were followed. At 21 days of age, 100 healthy birds with similar body weights were selected to reduce baseline variation and were randomly allocated to two treatment groups: a control group (CON), maintained at a constant temperature of 25 °C, and a heat stress group (HS), exposed to 35 °C from 08:00 to 20:00 daily, with the remaining time maintained under the same conditions as the CON group. This HS regimen has been repeatedly used and validated in our research group and was considered a reliable model of chronic HS. Each group consisted of five replicates, with 10 birds per replicate. Throughout the experiment, all birds had ad libitum access to a standard diet and water. The composition of the basal diet is shown in Table 1. Each replicate was considered an experimental unit for growth performance analysis, and one bird from each replicate was randomly selected at each sampling time point for tissue and serum analyses. All experimental procedures were approved by the Animal Ethics Committee of Henan University of Science and Technology (Approval No. DK20250725).

**Table 1:** Composition of ration and nutritional content.

Raw Material (%)	Day of Age	
	0–21 Days of Age	22–42 Days of Age
Maize	56–62	62–67
Soybean oil	2–4	3–6
Soybean meal	31–36	22–27
Dicalcium phosphate	1.5–1.8	1.0–1.4
Limestone	1.2–1.5	1.3–1.7
DL-methionine	0.20–0.35	0.10–0.22
L-lysine hydrochloride	0.15–0.28	0.08–0.18
Salt	0.2–0.3	0.2–0.3
Premix	1–3	1–3
Enzyme preparation	0.25	0.25
Probiotics	0.25	0.25

### 2.2. Sample Collection

Samples were collected at 28, 35, and 42 days of age. At each sampling time point, one bird from each replicate was randomly selected for routine sample collection. After euthanasia, blood samples were collected and centrifuged to obtain serum. The brain was then dissected using sterile instruments and rinsed with physiological saline to remove residual blood. A portion of the brain tissue was fixed in 4% paraformaldehyde for histopathological analysis, whereas the remaining tissue was rapidly frozen in liquid nitrogen and stored at −80 °C for subsequent analyses.

For the Evans blue extravasation assay, a separate bird from each replicate was randomly selected one day before each sampling time point. These birds were intravenously injected with 2% Evans blue solution (3.5 mL/kg). After 24 h, the birds were deeply anesthetized and subjected to cardiac perfusion, after which the brain tissue was rapidly collected, frozen in liquid nitrogen, and stored at −80 °C for further analysis. The Evans blue assay was performed on a different set of birds from those used for the other serum and tissue analyses.

### 2.3. Growth Performance Measurement

During the experiment, the average daily feed intake (ADFI) of each group was recorded. After a 12 h fasting period the day before sampling, the broilers were weighed in the morning at 8:00 on days 21 and 42, and the following formulas were used to calculate the performance parameters:

$$ADFI\;(g/bird/day)=\;\frac{Total\;feed\;intake}{Number\;of\;birds\;\times \;Experimental\;days}$$

$$ADG\;(g/bird/day)\;=\;\frac{Final\;body\;weight\;-\;Initial\;body\;weight}{Experimental\;days}$$

$$F/G\;(Feed-to-gain\;ratio)\;=\;\frac{\;average\;daily\;feed\;intake}{average\;daily\;gain}$$

### 2.4. Pathological Analysis

#### 2.4.1. Hematoxylin and Eosin (H&E) Staining

Brain tissue samples were collected from the same brain region in all broilers. After fixation in 4% paraformaldehyde, the tissues were routinely dehydrated, cleared, embedded in paraffin, and sectioned at a thickness of 4–6 μm. The sections were stained with hematoxylin and eosin (H&E), and histopathological changes were observed under a light microscope. To minimize observer bias, the histological evaluation was conducted in a blinded manner by investigators who were unaware of the group assignments.

#### 2.4.2. Transmission Electron Microscopy

Brain tissue samples were collected from the same brain region in all broilers and cut into small pieces. The tissues were initially fixed in electron microscopy fixative and subsequently post-fixed in 1% osmium tetroxide. After dehydration in a graded ethanol series, the samples were embedded in epoxy resin. Ultrathin sections (80 nm) were prepared, double-stained with uranyl acetate and lead citrate, and observed using a Hitachi HT7800 transmission electron microscope (HITACHI, Japan, Tokyo) at an accelerating voltage of 80.0 kV. Images were captured at a magnification of 50,000× for ultrastructural evaluation. Five fields per sample were analyzed for the ultrastructural evaluation.

### 2.5. Evans Blue Dye Extravasation Assay

Brain tissue samples (100 mg) from broilers were homogenized in 1 mL formamide solution. After centrifugation, the absorbance of the supernatant was measured at wavelengths of 620 nm and 740 nm. The dual-wavelength difference (ΔOD = OD_620_ − OD_740_) was calculated to eliminate background interference. The ΔOD value was then substituted into the standard curve equation, generated using serial dilutions of Evans blue standard solution, to determine the concentration of Evans blue in the samples.

### 2.6. Serum Biochemical Parameter Analysis

The activities of superoxide dismutase (SOD), glutathione peroxidase (GSH-Px), and catalase (CAT), as well as the levels of T-AOC and malondialdehyde (MDA) in serum samples, were measured using the corresponding kits from the Nanjing Jiancheng Bioengineering Institute. All procedures were performed strictly according to the instructions provided with the kits.

### 2.7. Quantitative Real-Time Polymerase Chain Reaction Assay

Gene expression in the brain tissue was measured as follows: the total RNA was extracted using TRIzol reagent, and the RNA quality was assessed prior to reverse transcription. Qualified RNA samples were diluted to the same concentration and reverse transcribed into cDNA. Before the formal experiment, the expression stability of β-actin was evaluated and confirmed to be suitable as a reference gene under the present experimental conditions. Therefore, β-actin was used as the internal control. The expression of HSP27, HSP70, HSP90α, TLR4, MYD88, NF-κB, NLRP3, ASC, Caspase-1, IL-1β, TNF-α, IL-4, NOX2, Iba1, iNOS, ZO-1, and Claudin-5 was quantified using SYBR Green qPCR. The relative gene expression was calculated using the 2^−∆∆Ct^ method. All qPCR reactions were performed in triplicate technical replicates. The primer sequences are listed in Table 2.

### 2.8. Statistical Analysis

All data were analyzed using IBM SPSS Statistics 27.0, and the graphs were prepared using GraphPad Prism 10.1.2. The data are expressed as mean ± SE. In the present study, the primary statistical objective was to compare the CON and HS groups within each specific age; therefore, analyses were conducted separately at each sampling time point. Differences between the two groups at each age were analyzed using independent-sample *t*-tests. Normality was assessed using the Shapiro–Wilk test, and the homogeneity of variance was evaluated using Levene’s test. Statistical significance was defined as *p* < 0.05.

## 3. Results

### 3.1. Growth Performance

To investigate the effects of chronic heat stress on growth performance in broilers, we measured the body weight of broilers from the CON group and the HS group at 21 and 42 days of age. As shown in Table 3, at 42 days of age, the HS group exhibited significantly lower body weight, ADG, and ADFI compared to the CON group (*p* < 0.01), while the F/G was significantly higher (*p* < 0.01).

### 3.2. Histopathological Changes in the Brain

**Figure 1:**
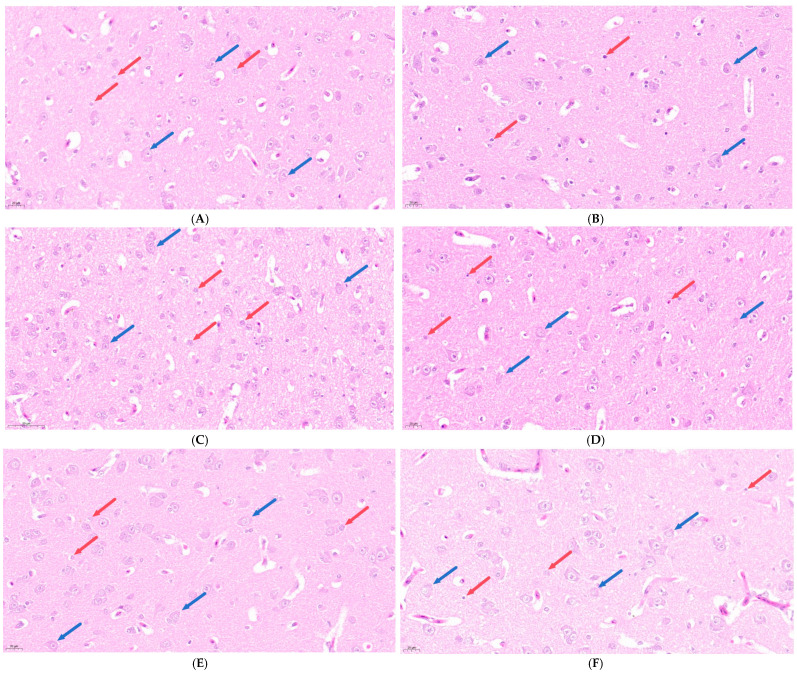
Effects of chronic HS on histopathological changes in the brain tissue of broilers (400× magnification). (**A**) CON group at 28 days of age; (**B**) HS group at 28 days of age; (**C**) CON group at 35 days of age; (**D**) HS group at 35 days of age; (**E**) CON group at 42 days of age; and (**F**) HS group at 42 days of age. Blue arrows indicate neurons and red arrows indicate glial cells.

Hematoxylin and eosin (H&E) staining was used to observe the pathological changes in the brain tissue of broilers under chronic HS, and the results are shown in Figure 1. At 28, 35, and 42 days of age, the HS group exhibited varying degrees of nuclear pyknosis, karyolysis, and cytolysis in neurons and glial cells compared to the CON group.

**Figure 2:**
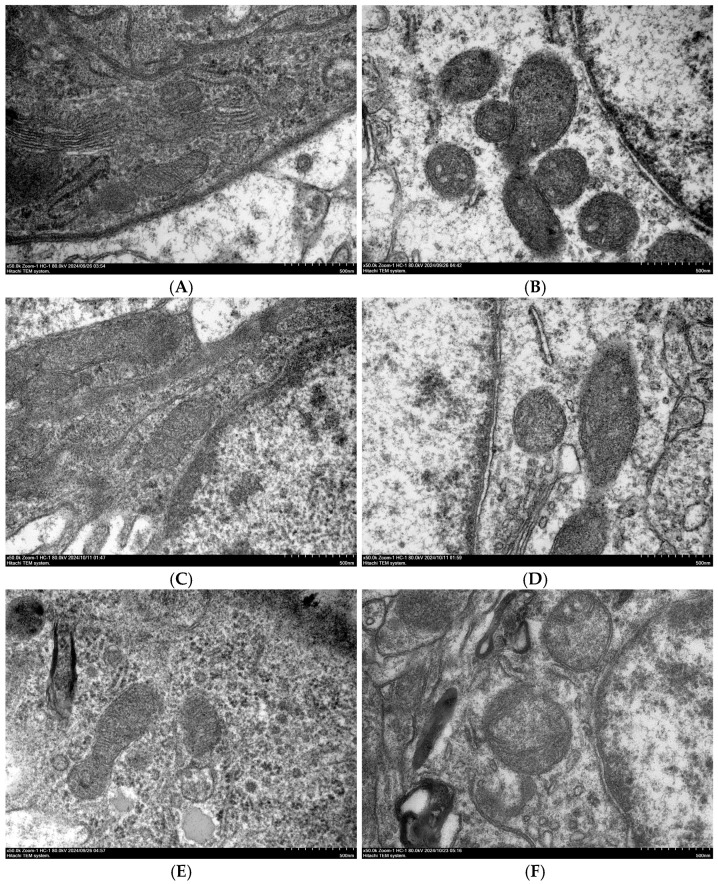
Ultrastructure of brain cells in broilers observed by transmission electron microscopy (50,000× magnification). (**A**) CON group at 28 days of age; (**B**) HS group at 28 days of age; (**C**) CON group at 35 days of age; (**D**) HS group at 35 days of age; (**E**) CON group at 42 days of age; and (**F**) HS group at 42 days of age.

Transmission electron microscopy (TEM) was used to examine the ultrastructure of brain cells, and the results are shown in Figure 2. At 28, 35, and 42 days of age, the HS group showed mitochondrial swelling compared to the CON group.

**Figure 3:**
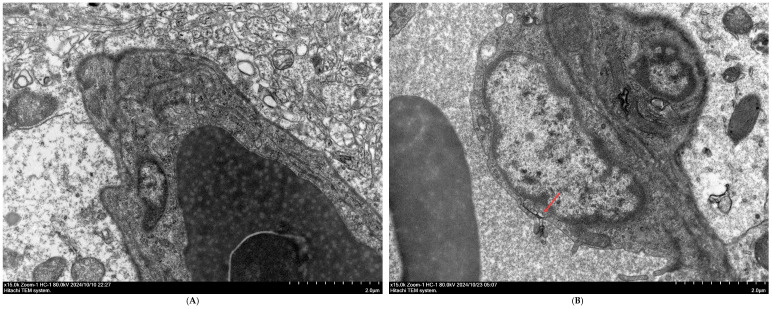
Ultrastructure of the BBB in broilers observed by TEM (15,000× magnification). (**A**) CON group at 42 days of age and (**B**) HS group at 42 days of age. The red arrow indicates the site of blood–brain barrier damage.

TEM was also used to observe the ultrastructure of the BBB at 42 days of age, and the results are shown in Figure 3. Compared to the CON group, the HS group exhibited evident damage to the BBB.

### 3.3. Evans Blue Dye Extravasation

**Figure 4:**
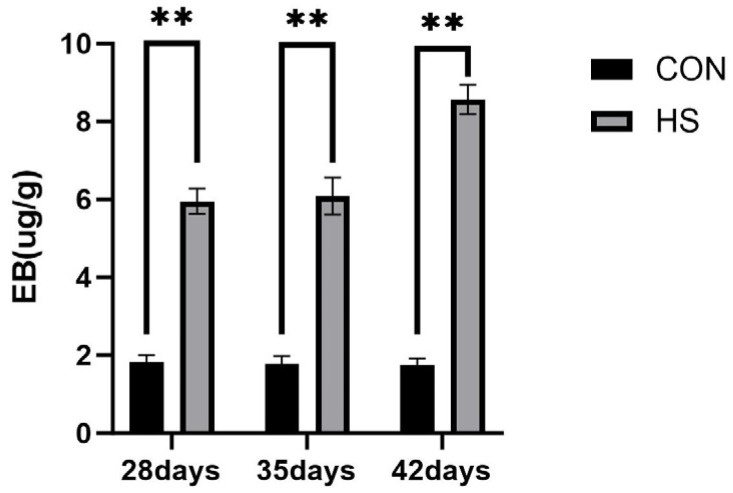
Results of Evans blue dye extravasation assay. Data are presented as mean ± SE (*n* = 5). CON: control group and HS: heat stress group. Differences were considered statistically significant at *p* < 0.05. **: *p* < 0.01.

Evans blue dye extravasation in the brain tissue at 28, 35, and 42 days of age is shown in Figure 4. At all three ages, the EB concentration was higher in the HS group than in the CON group (*p* < 0.01).

### 3.4. Serum Biochemical Parameters

**Figure 5:**
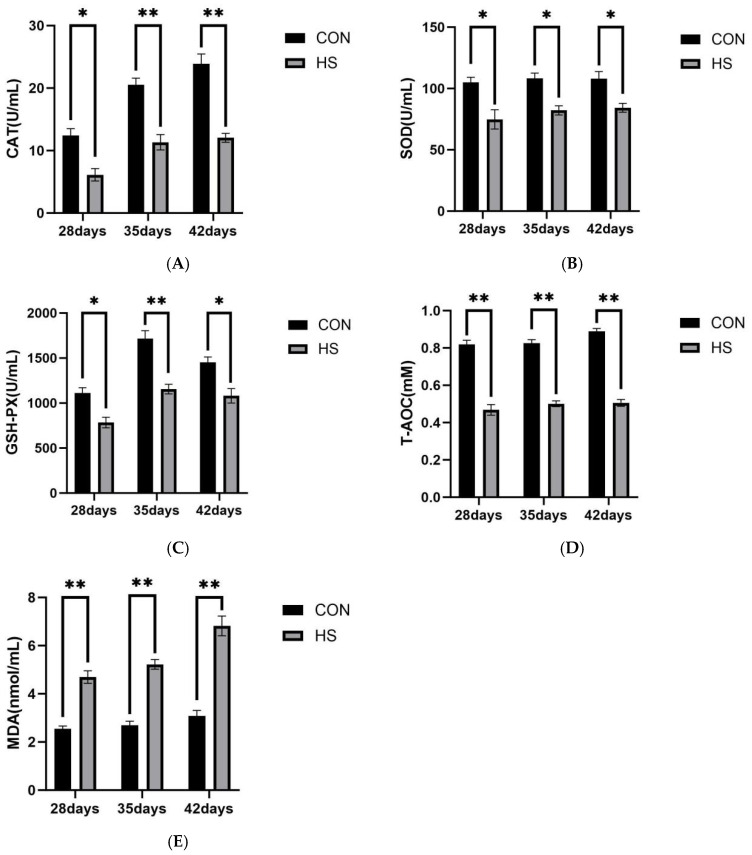
Results of serum biochemical analysis. (**A**) CAT, (**B**) SOD, (**C**) GSH-PX, (**D**) T-AOC, and (**E**) MDA. Data are presented as mean ± SE (*n* = 5). CON: control group and HS: heat stress group. Differences were considered statistically significant at *p* < 0.05. *: *p* < 0.05 and **: *p* < 0.01.

The serum levels of CAT (A), SOD (B), GSH-Px (C), T-AOC (D), and MDA (E) in broilers at 28, 35, and 42 days of age are shown in Figure 5.

At 28 days of age, the HS group showed lower levels of CAT, SOD, and GSH-Px (*p* < 0.05), as well as lower T-AOC and higher MDA levels (*p* < 0.01), compared with the CON group.

At 35 days of age, the CAT, GSH-Px, and T-AOC levels were lower in the HS group than in the CON group (*p* < 0.01), while SOD was also reduced (*p* < 0.05). In contrast, MDA was increased in the HS group (*p* < 0.01).

At 42 days of age, the HS group exhibited lower CAT and T-AOC levels (*p* < 0.01), together with reduced SOD and GSH-Px levels (*p* < 0.05), whereas MDA remained higher than that in the CON group (*p* < 0.01).

### 3.5. Relative mRNA Expression of Genes in the Brain

#### 3.5.1. Heat Shock Proteins

**Figure 6:**
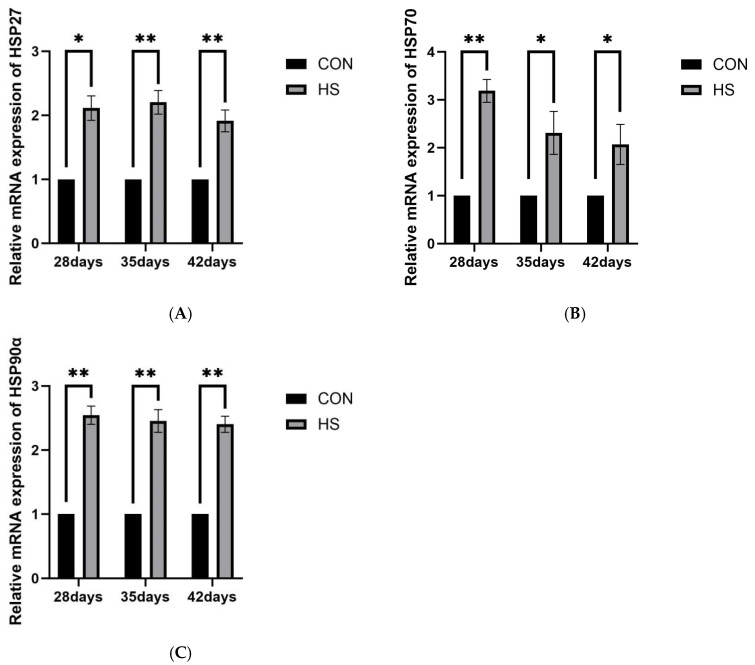
Effects of chronic HS on mRNA expression of heat shock protein genes in the brain of broilers. (**A**) HSP27, (**B**) HSP70, and (**C**) HSP90α. Data are presented as mean ± SE (*n* = 5). CON: control group and HS: heat stress group. Differences were considered statistically significant at *p* < 0.05. *: *p* < 0.05 and **: *p* < 0.01.

The relative mRNA expression levels of HSP27, HSP70, and HSP90α in brain tissue are shown in Figure 6. At 28 days of age, the HS group showed higher mRNA expression of HSP27 (*p* < 0.05) and HSP70 and HSP90α (*p* < 0.01) compared with the CON group. At 35 days of age, HSP27, HSP70, and HSP90α expression remained significantly elevated in the HS group (*p* < 0.05 for HSP70; *p* < 0.01 for HSP27 and HSP90α). At 42 days of age, HSP27 and HSP90α expression continued to be higher in the HS group (*p* < 0.01), whereas HSP70 expression was also increased (*p* < 0.05).

#### 3.5.2. Inflammatory Pathway-Related Genes

**Figure 7:**
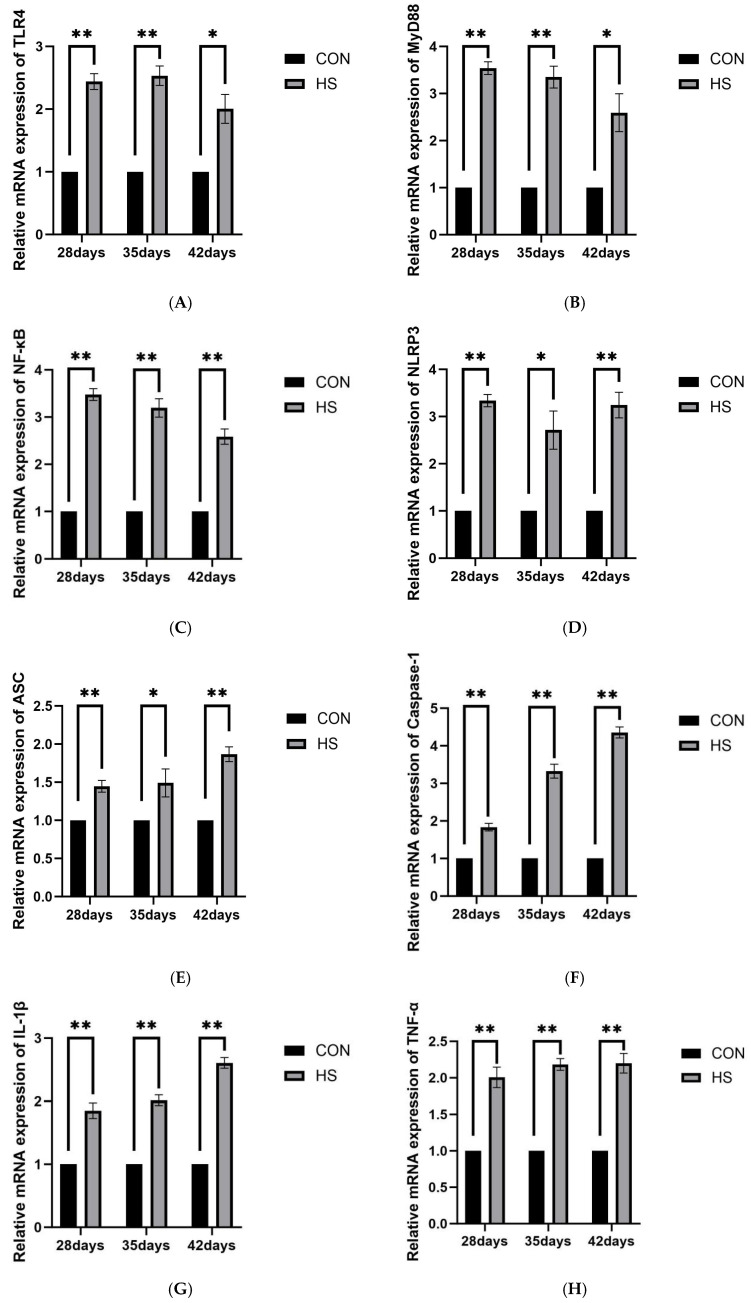

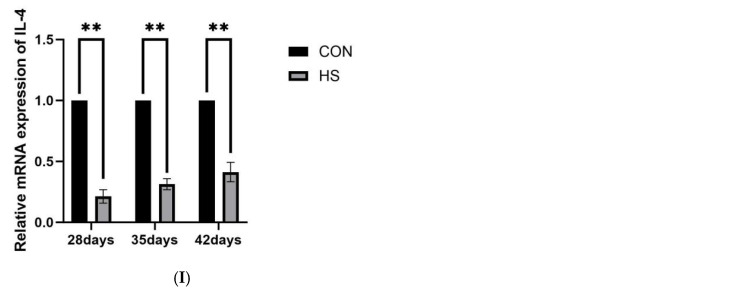
Effects of chronic HS on mRNA expression of inflammatory pathway-related genes in the brain of broilers. (**A**) TLR4, (**B**) MyD88, (**C**) NF-κB, (**D**) NLRP3, (**E**) ASC, (**F**) Caspase-1, (**G**) IL-1β, (**H**) TNF-α, and (**I**) IL-4. Data are presented as mean ± SE (*n* = 5). CON: control group and HS: heat stress group. Differences were considered statistically significant at *p* < 0.05. *: *p* < 0.05 and **: *p* < 0.01.

The relative mRNA expression levels of TLR4, MyD88, NF-κB, NLRP3, ASC, Caspase-1, IL-1β, TNF-α, and IL-4 are shown in Figure 7. At 28 days of age, the HS group exhibited significantly higher expression of TLR4, MyD88, NF-κB, NLRP3, ASC, Caspase-1, IL-1β, and TNF-α (*p* < 0.01), whereas IL-4 expression was lower (*p* < 0.01). At 35 days of age, the expression of TLR4, MyD88, NF-κB, Caspase-1, IL-1β, and TNF-α remained significantly higher in the HS group (*p* < 0.01), while NLRP3 and ASC were also elevated (*p* < 0.05); IL-4 expression remained reduced (*p* < 0.01). At 42 days of age, NF-κB, NLRP3, ASC, Caspase-1, IL-1β, and TNF-α expression was higher in the HS group (*p* < 0.01), TLR4 and MyD88 were also increased (*p* < 0.05), and IL-4 expression remained lower (*p* < 0.01).

#### 3.5.3. Oxidative Stress and Microglial Markers

**Figure 8:**
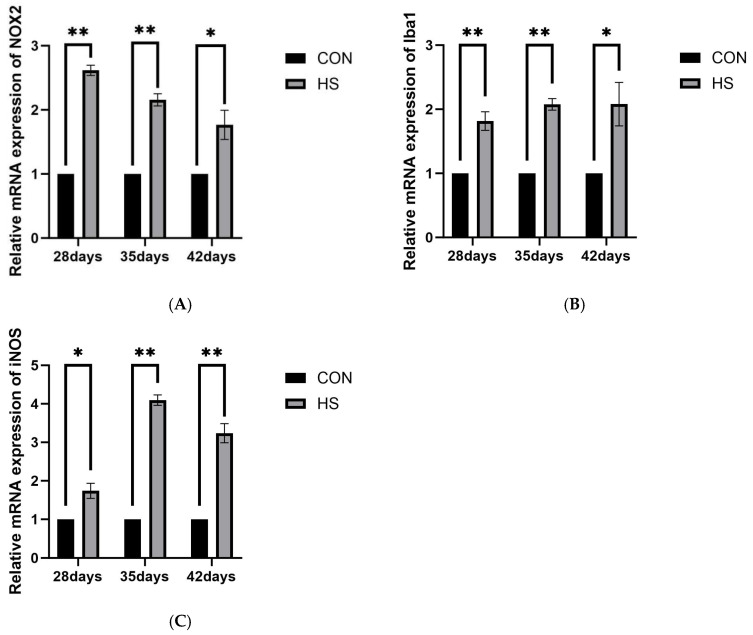
Effects of chronic HS on mRNA expression of oxidative stress and microglial marker genes in the brain of broilers. (**A**) NOX2, (**B**) Iba1, and (**C**) iNOS. Data are presented as mean ± SE (*n* = 5). CON: control group and HS: heat stress group. Differences were considered statistically significant at *p* < 0.05. *: *p* < 0.05 and **: *p* <0.01.

The relative mRNA expression levels of NOX2, Iba1, and iNOS are shown in Figure 8. At 28 days of age, the HS group showed higher expression of NOX2 and Iba1 (*p* < 0.01) and iNOS (*p* < 0.05). At 35 days of age, NOX2, Iba1, and iNOS expression was significantly higher in the HS group (*p* < 0.01). At 42 days of age, NOX2 and Iba1 remained elevated (*p* < 0.05), whereas iNOS expression was significantly higher in the HS group (*p* < 0.01).

### 3.6. mRNA Expression of Tight Junction-Related Proteins

**Figure 9:**
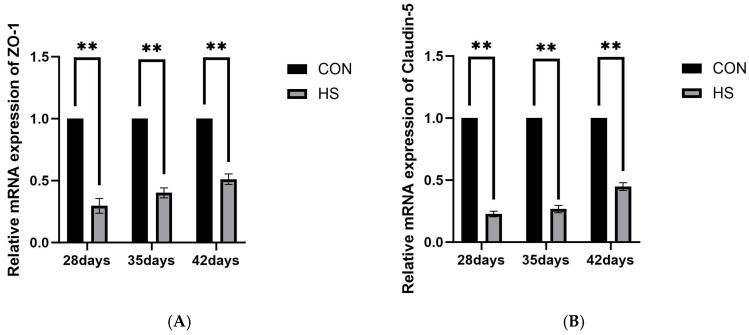
Effects of chronic HS on mRNA expression of tight junction-related genes in the brain of broilers. (**A**) ZO-1 and (**B**) Claudin-5. Data are presented as mean ± SE (*n* = 5). CON: control group and HS: heat stress group. Differences were considered statistically significant at *p* < 0.05. **: *p* < 0.01.

The mRNA expression of tight junction-related proteins ZO-1 (Figure 9A) and Claudin-5 (Figure 9B) in brain tissue was assessed at 28, 35, and 42 days of age by using quantitative PCR, as shown in Figure 9. The expression of ZO-1 and Claudin-5 was significantly lower in the HS group at all three ages compared to the CON group (*p* < 0.01).

## 4. Discussion

As global climate change intensifies, HS has become one of the major environmental factors affecting broiler production [41]. Due to their limited thermoregulatory capacity, broilers are highly susceptible to HS, which not only disrupts the heat dissipation balance but also triggers widespread stress responses and tissue damage [42]. The brain, as a critical regulatory center, particularly for thermoregulation, is especially sensitive to HS. Its high metabolic rate, high oxygen consumption, and abundance of polyunsaturated fatty acids make brain tissue more prone to oxidative damage and inflammation under HS conditions [43]. In the present study, chronic HS was associated with impaired growth performance, histopathological brain injury, oxidative imbalance, increased expression of inflammation-related genes, and BBB dysfunction in broilers, suggesting that these alterations may be closely linked during HS-associated brain injury.

This study first confirmed the negative impact of chronic HS on broiler growth performance. Compared with the CON group, the HS group showed reduced body weight, ADFI, and ADG, together with an increased F/G. These findings are consistent with previous studies showing that HS causes metabolic disturbances and redirects energy toward basic physiological maintenance, rather than growth [44]. Histopathological observations further revealed substantial brain injury. As early as 28 days of age, neuronal and glial cells in the HS group exhibited pathological changes, including nuclear pyknosis and karyolysis. Ultrastructural observations also showed mitochondrial swelling and BBB damage, indicating that chronic HS not only affects brain cell morphology but may also impair organelle function and barrier integrity.

Growing evidence indicates that OS and neuroinflammation are closely interconnected in heat-related brain injury [45,46,47]. In the present study, reduced serum antioxidant enzyme activities and T-AOC, together with increased MDA levels, indicated impaired systemic antioxidant defense and persistent OS. Similar associations between systemic stress and oxidative imbalance have also been reported in other veterinary settings [48]. In brain tissue, increased NADPH oxidase 2 (NOX2) mRNA expression suggested enhanced local ROS-generating potential [49,50]. Excessive ROS can directly damage lipids, proteins, and nucleic acids and may also act as danger signals that promote innate immune responses [51,52]. Elevated HSP70 and HSP90α expression further suggested a sustained stress response and the possible involvement of DAMP-related signaling [53]. In addition, increased ionized calcium-binding adapter molecule 1 (Iba1) expression, together with the upregulation of TLR4, MyD88, and NF-κB, suggests that microglia may participate in TLR4/MyD88/NF-κB-related inflammatory responses [54]. NF-κB may further promote the transcription of pro-inflammatory genes such as TNF-α, IL-1β, NLRP3, and ASC [55]. Previous studies have also implicated HSP90 in microglial pro-inflammatory responses [16]. In our study, the similar expression patterns of HSP90α and inducible nitric oxide synthase (iNOS), together with reduced IL-4 expression, suggest that chronic HS may shift the cerebral inflammatory milieu toward a more pro-inflammatory state.

The NLRP3 inflammasome-related pathway is widely considered an important component of inflammatory amplification [56]. Its activation generally requires NF-κB-mediated transcriptional priming, together with intracellular danger signals such as ROS [57,58]. In the present study, NLRP3, ASC, Caspase-1, and IL-1β mRNA expression remained elevated under HS, particularly at 35 days of age, when NOX2 and iNOS-related responses were also more pronounced. These findings suggest that NLRP3 inflammasome-related signaling may be enhanced at the transcriptional level during chronic HS. However, because the present study was based primarily on qPCR data, no direct evidence was obtained for inflammasome assembly, Caspase-1 cleavage, or pyroptotic execution. Therefore, the mechanistic interpretation of this pathway should be made cautiously.

The inflammatory changes observed in the present study were also associated with BBB impairment. Evans blue extravasation showed increased vascular permeability in the HS group at 28, 35, and 42 days of age, indicating sustained BBB dysfunction. Meanwhile, ZO-1 and Claudin-5 mRNA expression was significantly reduced. Because BBB integrity depends critically on tight-junction proteins such as ZO-1 and Claudin-5 [59], these findings are consistent with BBB impairment. In addition, pro-inflammatory cytokines such as TNF-α and IL-1β have been reported to suppress tight-junction protein expression and alter their localization, thereby weakening the BBB function [60]. Taken together, these results suggest that enhanced inflammatory responses under HS may contribute to BBB structural and functional impairment. Nevertheless, because no protein-level or localization analysis of tight-junction proteins was performed, conclusions regarding BBB molecular alterations should also be interpreted cautiously.

Several limitations of the present study should be acknowledged. First, the mechanistic interpretation relied mainly on mRNA expression changes, without protein-level validation of TLR4/MyD88/NF-κB/NLRP3-related signaling or functional assessment of inflammasome activation and microglial status. Second, BBB-related conclusions were based on Evans blue extravasation and tight junction-related mRNA expression, whereas protein localization and quantitative ultrastructural analyses were not performed. Third, the statistical analyses were mainly conducted as age-stratified comparisons between the CON and HS groups; accordingly, treatment × age interaction was not formally evaluated, and temporal interpretations should be made cautiously. Fourth, due to the limitations of the experimental conditions, the sample size was five birds per group per time point. This limitation should be taken into account when interpreting the results.

In summary, through comparison between the CON and HS groups, the present study provides evidence that chronic HS in broilers is associated with systemic oxidative imbalance, inflammation-related gene expression changes, and BBB impairment. Rather than demonstrating a definitive causal pathway, our findings support a potential pathological framework in which OS, neuroinflammatory responses, and BBB dysfunction may interact and collectively aggravate brain injury, with the changes between 28 and 35 days of age suggesting a potentially more vulnerable stage during chronic HS exposure.

## 5. Conclusions

In conclusion, chronic HS was associated with marked pathological injury in the broiler brain, accompanied by reduced growth performance, impaired antioxidant capacity, increased expression of inflammation-related genes, and BBB dysfunction. The present findings suggest that HS-related brain injury in broilers is closely associated with enhanced OS, transcriptional upregulation of TLR4/MyD88/NF-κB/NLRP3 pathway-related genes, and impaired BBB integrity. These alterations may not occur independently, but rather may interact and collectively contribute to the progression of brain injury.

In addition, the changes observed between 28 and 35 days of age suggest that this period may represent a potentially more vulnerable stage during chronic HS exposure, as reflected by relatively more pronounced inflammatory changes and tissue injury. However, this temporal pattern should be interpreted cautiously. Although the magnitude of some upstream changes became less marked by 42 days of age, the inflammation-related alterations and BBB impairment remained evident, suggesting that brain injury may persist under prolonged HS conditions. Therefore, early environmental regulation and nutritional intervention before or during this period may help to alleviate the adverse effects of HS on brain health and production performance in broilers.

## Figures and Tables

**Table 2:** Information for sequences of the oligonucleotide primers.

Gene	Sequences (5′-3′)
β-actin F	CCGCTCTATGAAGGCTACGC
β-actin R	CTCTCGGCTGTGGTGGTGAA
HSP27 F	ACGTCAACCACTTTGCTCCT
HSP27 R	TATTTGCGGGTGAAGCACCT
HSP70 F	TGTGGCCTTCACCGATACAG
HSP70 R	TGGGGTCATCATACTTGCGG
HSP90α F	AATGGAGGAGGAAGTGGAGACC
HSP90α R	CAGAGTGCGATCATGCTTGTTT
TLR4 F	CCAAACACCACCCTGGACTT
TLR4 R	CCATGGAAGGCTGCTAGACC
MyD88 F	GAGGGATGATCCGTATGGGC
MyD88 R	ACACGTTCCTGGCAAGACAT
NF-κB F	ACACCACTGGATATGGCAGC
NF-κB R	TCTTGCTTGGATCAGGCGTT
NLRP3 F	TAGAGTACGCGGGTGAAGGA
NLRP3 R	CTGTGAAACTGCCCAACACG
ASC F	AAGCTGCTGCGTAAGAAGGA
ASC R	CGTAGGTGCTGCTTGTTGTC
Caspase-1 F	GCTGGACCTCAGAGGAACCA
Caspase-1 R	TCGCATCGTCATCTTCACCT
IL-1β F	TTGAGCCCGTCACCTTCCA
IL-1β R	CAATGTTGAGCCTCACTTTCTGG
TNF-α F	GCTGACGGTGGACCTATTATTGTAGAG
TNF-α R	TTCTTCACGCCATCAGGAAGGTTG
IL-4 F	TGCTTACAGCTCTCAGTGCC
IL-4 R	TCTTGACGCAGGAAACCTCTC
NOX2 F	TGGCTGGTCTCACTGGTGTTAT
NOX2 R	CCTTTCTTTCCCCAGTCAGTGAA
Iba1 F	GCTGGATGGCTTAAGACAGG
Iba1 R	AGAGCAACAGATAAGGCGCA
iNOS F	AAGCAAGCCCTCACCTACCT
iNOS R	CCATCCTCCACGATCCTGTA
ZO-1 F	CTTCAGGTGTTTCTCTTCCTCCTC
ZO-1 R	CTGTGGTTTCATGGCTGGATC
Claudin-5 F	CATCACTTCTCCTTCGTCAGC
Claudin-5 R	GCACAAAGATCTCCCAGGTC

**Table 3:** Effects of chronic heat stress on growth performance in broilers.

Growth Performance	Group		*p*-Value
	CON	HS	
Weight at 21 days of age (g)	893.1 ± 37.2	901.5 ± 35.6	0.853
Weight at 42 days of age (g)	2604.9 ± 30.6	1926.7 ± 41.3	<0.01
ADG (g)	81.49 ± 2.62	48.65 ± 2.93	<0.01
ADFI (g)	133.44 ± 2.33	117.93 ± 4.51	<0.01
F/G	1.63 ± 0.037	2.42 ± 0.044	<0.01

Data are presented as mean ± SE (*n* = 5). CON: control group and HS: heat stress group. Differences were considered statistically significant at *p* < 0.05.

## Data Availability

The original contributions presented in this study are included in the article. Further inquiries can be directed to the corresponding author.
